# One-Dimensional Theoretical Solution and Two-Dimensional Numerical Simulation for Functionally-Graded Piezoelectric Cantilever Beams with Different Properties in Tension and Compression

**DOI:** 10.3390/polym11111728

**Published:** 2019-10-23

**Authors:** Xiao-Ting He, Zhi-Xin Yang, Hong-Xia Jing, Jun-Yi Sun

**Affiliations:** 1 School of Civil Engineering, Chongqing University, Chongqing 400045, China; 20141602063@cqu.edu.cn (Z.-X.Y.); jinghongxiajy@163.com (H.-X.J.); sunjunyi@cqu.edu.cn (J.-Y.S.); 2 Key Laboratory of New Technology for Construction of Cities in Mountain Area (Chongqing University), Ministry of Education, Chongqing 400045, China

**Keywords:** functionally graded piezoelectric materials, bimodulus, cantilever beams, tension and compression, neutral layer

## Abstract

The existing studies indicate polymers will present obviously different properties in tension and compression (bimodular effect) which is generally ignored because of the complexity of the analysis. In this study, a functionally graded piezoelectric cantilever beam with bimodular effect was investigated via analytical and numerical methods, respectively, in which a one-dimensional theoretical solution was derived by neglecting some unimportant factors and a two-dimensional numerical simulation was performed based on the model of tension-compression subarea. A full comparison was made to show the rationality of one-dimensional theoretical solution and two-dimensional numerical simulation. The result indicates that the layered model of tension-compression subarea also makes it possible to use numerical technique to simulate the problem of functionally graded piezoelectric cantilever beam with bimodular effect. Besides, the modulus of elasticity E^*^ and the bending stiffness D^*^ proposed in the one-dimensional problem may succinctly describe the piezoelectric effect on the classical mechanical problem without electromechanical coupling, which shows the advantages of one-dimensional solution in engineering applications, especially in the analysis and design of energy harvesting/sensing/actuating devices made of piezoelectric polymers whose bimodular effect is relatively obvious.

## 1. Introduction

Piezoelectric materials have an electromechanical coupling characteristic, which makes them a good candidate for a variety of electromechanical devices, for example, sensors and actuators used extensively in electromechanical conversion. Piezoelectric sensors are usually a laminated original made by ceramic slice, so it is easy to cause stress concentration and promote the growth of interfacial microcracks. In order to overcome this difficulty, scholars developed functionally graded piezoelectric materials (FGPM) whose properties of materials change continuously along certain direction. There is no obvious interface in FGPM, thus the damage due to the stress concentration at the interface is effectively avoided. Studies on FGPM have attracted the attention of scholars from all over the world. In recent years, with the development, universality and miniaturization of electronic devices, new piezoelectric materials continue to emerge, among which piezoelectric polymers play an increasingly important role [1,2,3,4]. Piezoelectric polymers are attractive for wearable due to their flexibility and conformability over piezoelectric ceramic materials. Studies of piezoelectric polymers have gradually become a hot topic for scholars. In this study, we will introduce the concept of bimodular materials into the above analyses. Thus, our work relates to three aspects of properties of materials, i.e., functionally graded, piezoelectric and bimodular, which are reviewed briefly as the following.

In the 1990s, the concept of functionally grade materials [5] was first introduced into the field of piezoelectric materials. From that time on, more scholars from all over the world have been involved in the study of FGPM and successfully fabricated FGPM sensors and actuators [6,7,8]. The stress concentration can be effectively avoided and also there is no paste interface by using FGPM to produce smart devices, which greatly increases the performance and life-time of smart devices. From the late twentieth century to the early twenty-first century, scholars made great efforts to achieve solutions of relating problems on piezoelectric beams and FGPM beams, thus accelerating the application of FGPM sensors and actuators in the field of engineering. Aimed at the shape control and deformation problems of beams with piezoelectric actuators, Wang et al. [9] and Yang and Ngoi [10] obtained the exact solutions and analytical expressions of the bending problems. Under various mechanical boundary conditions, Smits et al. [11] derived the constituent equations for piezoelectric bimorphs. By adopting Euler–Bernoulli beam theory, Elshafei et al. [12] made a static analysis for beams with piezoelectric actuators subjected to axial, transverse, and torsion loads, and derived the equation of motion of the structure system. Without specifying the function expressions of materials, Zhong and Yu [13,14] proposed the general elasticity solutions for FGPM beams subjected to various loads. Huang et al. [15,16] obtained two piezoelasticity solutions for FGPM cantilever beams under different loading conditions, and presented piezoelectricity solutions for the two actuators subjected to a constant voltage. Shi and Chen [17] studied the problem of a FGPM cantilever beam and obtained a set of analytical solutions for the beam subjected to different loadings. By studying the electrostatic problem of FGPM cantilevers, Xiang and Shi [18] suggested a general formula to calculate the tip deflection of a FGPM actuator when two types of plane problem (plane stress problem and plane strain problem) are not satisfied. Under different loading cases and different boundary conditions, Yao and Shi [19] studied the steady-state forced vibration of a FGPM beam. By proposing a solution modification of a piezoelectric bimorph cantilever under loads, Shi et al. [20] obtained the analytical solutions of a piezoelectric bimorph cantilever under three different loading conditions. There are many other topics on FGPM beams; due to the length limit of the paper, the review in detail is not presented here.

By comparison with the FGPM, bimodular material seems to be relatively less well-known. In the light of the classical theory of elasticity, elastic moduli in tension and compression are the same. However, many studies indicate that some materials [21,22], like ceramics, plastics, steel concrete, graphite, powder metallurgy materials, polymeric materials, and some composites, will present different elastic properties when they are stretched and compressed, that is, they have different moduli in tension and compression and thus are referred to as bimodular materials. In 1982, Ambartsumyan [23] published the first monograph titled by Elasticity Theory of Different Moduli, in which the constitutive model of bimodular materials and corresponding structural analysis based on the materials model are systematically introduced. In this monograph, Ambartsumyan pointed out that some structural materials including reinforced and unreinforced polymers have considerably strong bimodular characteristics. The bimodular characteristics of polymers is mainly concerned with the manufacturing process, masses of contact materials and reinforced materials, as well as temperature. The publication of this book marks that the idea of bimodular materials has entered the field of vision of scholars. Thereafter, bimodular problems concerning materials and structures have been extensively studied [24,25,26]. These researches indicate that the introduction of bimodular effect of materials will modify the mechanical properties of structures to some extent. Unfortunately, due to the complexity of its analysis, the bimodular effect of materials is often neglected, especially in the analysis of some specialized materials and structures, for example, intelligent materials and structures mentioned above.

More recently, He et al. [27] introduced the bimodular effect into the analysis of FGPM structures, for the first time, and obtained a two-dimensional electroelastic analytical solution for a FGPM beam with different moduli in tension and compression. Although the effectiveness of the analytical work was verified by comparing with the existing theoretical work presented by Zhong and Yu [13], it is also a pity that the comparison was based on the degraded analytic expressions from He et al. [27] since the work of Zhong and Yu [13] did not consider the bimodular effect. Now that existing analytical works are not fully qualified for this comparative work, we have to resort to the numerical technique based on existing software such as ABAQUS. For a traditional piezoelectric problem, the use of ABAQUS appears to be only a step-by-step process. However, how about after introducing the bimodular effect and functionally graded properties of the materials? In addition, similar to the bending problem of classical beams, a so-called one-dimensional solution and two-dimensional solution under different application conditions always exists. Now that the two-dimensional theoretical solution for a bimodular FGPM beam can be obtained [27], what is the form of one-dimensional solution? For the above two reasons, we think it is necessary to obtain the one-dimensional theoretical solution and also perform the two-dimensional numerical simulation for the problem of bimodular FGPM cantilever beam. Therefore, this study may serve as a new supplement to the existing works, not only from the theoretical aspect but also from the point of view of numerical simulation.

In this study, we will derive one-dimensional theoretical solution by neglecting some unimportant factors and perform two-dimensional numerical simulation based on the model of tension-compression subarea. The whole paper is organized as follows. In Section 2, the solving problem will be described, including the definition of different properties in tension and compression and the constitutive equation of FGPM in a two-dimensional case. The one-dimensional theoretical solution will be derived in Section 3 and the two-dimensional numerical simulation will be performed in Section 4. Next, in Section 5, we will make extensive comparisons with existing studies which not only include previous studies of our themselves [27] but also the work from other authors, to show the validity of our work, and also study the evolution from classical beams to bimodular FGPM beams as well as discuss the deformation of flexible piezoelectric structures. According to the results allude to above, some main conclusions will be drawn in Section 6.

## 2. The Problem Description

As indicated above, the characteristics of material considered in this study include the bimodular effect, the functionally graded property and the piezoelectric characteristic. In practical applications, there is a large number of materials containing three properties above, for example, the functionally graded material made of piezoelectric ceramics and steel, at the same time, the bimodular effect is considered due to the existence of ceramics, which present relatively obvious different elastic properties in tension and compression (see [23]). Among these applications, they usually exist in a certain structural form, for example, the form of cantilever beam; thereby it is necessary to study a bimodular functionally graded piezoelectric cantilever beam.

**Figure 1:**
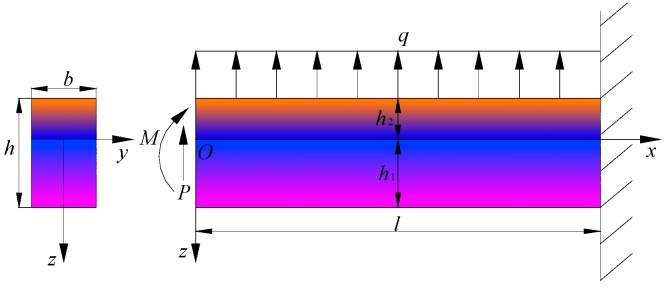
Scheme of a bimodular functionally graded piezoelectric materials (FGPM) cantilever beam under combined loads.

An orthotropic functionally graded piezoelectric cantilever beam with bimodular effect is considered here, as shown in Figure 1, in which the right end of the beam is fully fixed and the left end free; h×b stands for the rectangular section dimension of the beam and l is the length of the beam (h≪l). Without losing generality, the loads may be considered as single form load, for example, the upper layer of the beam is subjected to uniformly distributed load q, or the left end of the beam is subjected to a shear force P or a bending moment M, or the combined loads from above load forms, as shown in Figure 1. It is obvious that the loads acting in plane coordinate system *xoz* may cause downward bending of the beam, thereby generating the so-called tensile part and compressive part, bounded by the neutral layer. Thus, we establish a rectangular coordinate system in which z=0 is exactly at the neutral layer, as shown in Figure 1. The upper and lower edge layers are $z=-h_{2}$ and $z=h_{1}$, respectively, in which $h_{1}$ is the tensile height and $h_{2}$ the compressive height according to previous studies [28,29]. Note that due to the introduction of functionally graded property, physical parameters of materials of the beam are also functions of coordinates. Generally, it is assumed that physical parameters vary only along certain direction, for example, the thickness direction. In this study, material parameters are assumed to vary with *z*, in the light of the following rules

(1) $$\begin{array}{l} s_{ij}^{+}=s_{ij}^{0}F^{+}(z),d_{ij}^{+}=d_{ij}^{0}F^{+}(z),\lambda_{ij}^{+}=\lambda_{ij}^{0}F^{+}(z),\\ s_{ij}^{-}=s_{ij}^{0}F^{-}(z),d_{ij}^{-}=d_{ij}^{0}F^{-}(z),\lambda_{ij}^{-}=\lambda_{ij}^{0}F^{-}(z) \end{array}$$

where $F^{+}(z)=e^{\alpha_{1}z/h},F^{-}(z)=e^{\alpha_{2}z/h}$ are gradient functions in tension and compression, respectively; superscript “+” denotes a tensile quantity and “−” compressive quantity; $s_{ij}^{+/-},d_{ij}^{+/-},\lambda_{ij}^{+/-}$ are elastic coefficient, piezoelectric coefficient and dielectric coefficient, respectively; $s_{ij}^{0},d_{ij}^{0},\lambda_{ij}^{0}$ are values of corresponding material parameters at the neutral layer (z=0), respectively. Note that a set of very small electrodes are adhered discontinuously to the upper and lower surfaces of the beam, and at the same time the beam is poled along the direction of *z*. In two-dimensional problem, let the stress components and strain components be $\sigma_{x}^{+/-},\sigma_{z}^{+/-},\tau_{zx}^{+/-}$ and $\varepsilon_{x}^{+/-},\varepsilon_{z}^{+/-},\gamma_{zx}^{+/-}$, respectively; let the electrical displacement components and the electrical field components be $D_{x}^{+/-},D_{z}^{+/-}$ and $E_{x}^{+/-},E_{z}^{+/-}$, respectively, thus the physical equations give

(2) $$\left\{\begin{array}{l} \varepsilon_{x}^{+/-}\\ \varepsilon_{z}^{+/-}\\ \gamma_{zx}^{+/-} \end{array}\right\}=\left[\begin{array}{ccc} s_{11}^{+/-} & s_{13}^{+/-} & 0\\ s_{13}^{+/-} & s_{33}^{+/-} & 0\\ 0 & 0 & s_{44}^{+/-} \end{array}\right]\left\{\begin{array}{l} \sigma_{x}^{+/-}\\ \sigma_{z}^{+/-}\\ \tau_{zx}^{+/-} \end{array}\right\}+\left[\begin{array}{cc} \begin{array}{l} 0\\ 0\\ d_{15}^{+/-} \end{array} & \begin{array}{l} d_{31}^{+/-}\\ d_{33}^{+/-}\\ 0 \end{array} \end{array}\right]\left\{\begin{array}{l} E_{x}^{+/-}\\ E_{z}^{+/-} \end{array}\right\}$$

and

(3) $$\left\{\begin{array}{l} D_{x}^{+/-}\\ D_{z}^{+/-} \end{array}\right\}=\left[\begin{array}{ccc} \begin{array}{l} 0\\ d_{31}^{+/-} \end{array} & \begin{array}{l} 0\\ d_{33}^{+/-} \end{array} & \begin{array}{l} d_{15}^{+/-}\\ 0 \end{array} \end{array}\right]\left\{\begin{array}{l} \sigma_{x}^{+/-}\\ \sigma_{z}^{+/-}\\ \tau_{zx}^{+/-} \end{array}\right\}+\left[\begin{array}{cc} \lambda_{11}^{+/-} & 0\\ 0 & \lambda_{33}^{+/-} \end{array}\right]\left\{\begin{array}{l} E_{x}^{+/-}\\ E_{z}^{+/-} \end{array}\right\},$$

where superscript “+/−” still denotes a tensile (compressive) quantity, similar to Equation (1). It may be inferred that the constitutive relation in two-dimensional case may be moderately simplified in one-dimensional problem, according to our previous study concerning one-dimensional and two-dimensional problems of bimodular FGM beams [30].

## 3. One-Dimensional Theoretical Solution

**Figure 2:**
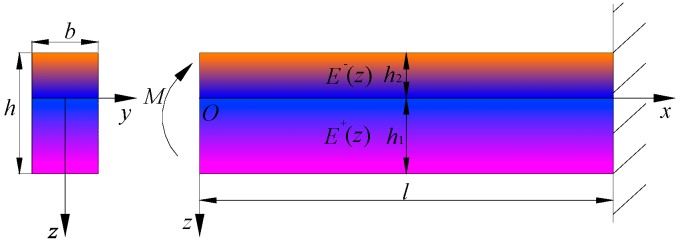
Scheme of a bimodular FGPM cantilever beam under pure bending.

In one-dimensional problem, we may consider the simplest case, i.e., the pure bending problem shown in Figure 2, in which the left end of the cantilever beam is subjected to a bending moment *M.* Obviously, the beam will generate downward bending under the action of the bending moment in plane coordinate system *xoz*, thus forming a so-called tensile zone and compressive zone. We still establish the neutral layer at z=0, and the tensile modulus of elasticity is denoted by $E^{+}(z)$ and the compressive one by $E^{-}(z)$; similarly, the tensile and compressive heights are $h_{1}$ and $h_{2}$, respectively, as shown in Figure 2.

### 3.1. Mechanical Stress and Deflection

Note that in such a one-dimensional pure-bending problem, there is no stresses $\sigma_{z}^{+/-}$ and $\tau_{zx}^{+/-}$ as well as the corresponding strain $\varepsilon_{z}^{+/-}$ and $\gamma_{zx}^{+/-}$, only existing $\sigma_{x}^{+/-}$ and $\varepsilon_{x}^{+/-}$, from the point of view of deformation. Thus, the constitutive relation of FGPM with different properties in tension and compression, i.e., Equations (2) and (3), may be simplified as

(4) $$\left\{ \begin{array}{l} \varepsilon_{x}^{+/-}=s_{11}^{+/-}\sigma_{x}^{+/-}+d_{31}^{+/-}E_{z}^{+/-}\\ \varepsilon_{z}^{+/-}=0\\ \gamma_{zx}^{+/-}=0 \end{array}\right.$$

and

(5) $$\left\{ \begin{array}{l} D_{x}^{+/-}=\lambda_{11}^{+/-}E_{x}^{+/-}\\ D_{z}^{+/-}=d_{31}^{+/-}\sigma_{x}^{+/-}+\lambda_{33}^{+/-}E_{z}^{+/-} \end{array}\right..$$

In existing studies for two-dimensional problem [13,14,27], $D_{x}>>D_{z}$ may be found, thus we may assume $D_{z}\approx 0$ in the one-dimensional problem. From Equation (5), we have

(6) $$E_{z}^{+/-}=-\frac{d_{31}^{+/-}}{\lambda_{33}^{+/-}}\sigma_{x}^{+/-}.$$

Substituting Equation (6) into Equation (4), we obtain

(7) $$\varepsilon_{x}^{+/-}=\left[\frac{s_{11}^{+/-}\lambda_{33}^{+/-}-{\left(d_{31}^{+/-}\right)}^{2}}{\lambda_{33}^{+/-}}\right]\sigma_{x}^{+/-}=\frac{\sigma_{x}^{+/-}}{E^{*}},$$

where $E^{*}$ is defined as the modulus of elasticity in one-dimensional problem, i.e.,

(8) $$E^{*}=\frac{\lambda_{33}^{+/-}}{s_{11}^{+/-}\lambda_{33}^{+/-}-{\left(d_{31}^{+/-}\right)}^{2}}.$$

We note that if the above equation is rewritten as the form $E^{*}={\left[s_{11}^{+/-}-{\left(d_{31}^{+/-}\right)}^{2}/\lambda_{33}^{+/-}\right]}^{-1}$, it may clearly explain the piezoelectric effect on the modulus of elasticity in classical problem. Specially, when $s_{11}^{+/-}>>{\left(d_{31}^{+/-}\right)}^{2}/\lambda_{33}^{+/-}$ (for example, for the piezoelectric materials PZT-4, it is the case), we may have $E^{*}=1/s_{11}^{+/-}$, which is exactly the reciprocal relationship between flexibility coefficient and stiffness coefficient. From the viewpoint of regression satisfaction, this fact verifies indirectly the correctness of Equations (4) and (5) in one-dimensional case. After substituting the functionally graded form, i.e., Equation (1), into Equation (7), we have

(9) $$\sigma_{x}^{+/-}=\frac{\lambda_{33}^{+/-}}{s_{11}^{+/-}\lambda_{33}^{+/-}-{\left(d_{31}^{+/-}\right)}^{2}}\varepsilon_{x}^{+/-}=\frac{\lambda_{33}^{0}}{s_{11}^{0}\lambda_{33}^{0}-{\left(d_{31}^{0}\right)}^{2}}\cdot \frac{1}{e^{\alpha_{i}z/h}}\varepsilon_{x}^{+/-},$$

where $e^{\alpha_{i}z/h}$ are gradient functions, introduced earlier in Equation (1), $\alpha_{i}(i=1,2)$ corresponds to “+/−”; and $s_{11}^{0}$,$\lambda_{33}^{0}$ and $d_{31}^{0}$ represent the values of material parameters $s_{11}$,$\lambda_{33}^{}$ and $d_{31}$ at the neutral layer (z=0), respectively.

If we let the curvature radius of the beam in bending be ρ, the strain at any point may be expressed as, according to plane section assumption in a one-dimensional case,

(10) $$\varepsilon_{x}^{+/-}=\frac{z}{\rho }.$$

Substituting Equation (10) into Equation (9), we obtain

(11) $$\sigma_{x}^{+/-}=\frac{\lambda_{33}^{0}}{s_{11}^{0}\lambda_{33}^{0}-{\left(d_{31}^{0}\right)}^{2}}\cdot \frac{z}{\rho \cdot e^{\alpha_{i}z/h}}=\frac{k_{0}}{\rho }\frac{z}{e^{\alpha_{i}z/h}},$$

where $k_{0}=\frac{\lambda_{33}^{0}}{s_{11}^{0}\lambda_{33}^{0}-{\left(d_{31}^{0}\right)}^{2}}$. Thus, we obtain the bending stress in tensile and compressive zone for the one-dimensional pure-bending problem, i.e., for $0\leq z\leq h_{1}$,

(12) $$\sigma_{x}^{+}=\frac{k_{0}}{\rho }ze^{-\alpha_{1}z/h},$$

and for $-h_{2}\leq z\leq 0$,

(13) $$\sigma_{x}^{-}=\frac{k_{0}}{\rho }ze^{-\alpha_{2}z/h}.$$

Note that ρ, $h_{1}$ and $h_{2}$, as well as the deflection of the beam are still not determined yet. Next, we will use the conditions of internal forces on the section to determine them.

Let the normal internal force acting on any section be *N*, thus N=0 will give

(14) $$\int_{0}^{h_{1}}\sigma_{x}^{+}bdz+\int_{-h_{2}}^{0}\sigma_{x}^{-}bdz=0.$$

Substituting Equations (12) and (13) into Equation (14), we have

(15) $$\int_{0}^{h_{1}}\frac{k_{0}b}{\rho }e^{-\alpha_{1}z/h}zdz+\int_{-h_{2}}^{0}\frac{k_{0}b}{\rho }e^{-\alpha_{2}z/h}zdz=0.$$

where $k_{0}b/\rho$ is a constant and may be deleted in the above equation. If we let

(16) $$\left\{ \begin{array}{l} \int_{0}^{h_{1}}e^{-\alpha_{1}z/h}zdz=e^{-\alpha_{1}h_{1}/h}\left(-\frac{hh_{1}}{\alpha_{1}}-\frac{h^{2}}{\alpha_{1}^{2}}\right)+\frac{h^{2}}{\alpha_{1}^{2}}=A_{1}^{+}\\ \int_{-h_{2}}^{0}e^{-\alpha_{2}z/h}zdz=e^{\alpha_{2}h_{2}/h}\left(-\frac{hh_{2}}{\alpha_{2}}+\frac{h^{2}}{\alpha_{2}^{2}}\right)-\frac{h^{2}}{\alpha_{2}^{2}}=A_{1}^{-} \end{array}\right.,$$

from Equation (15), we may obtain

(17) $$A_{1}^{+}+A_{1}^{-}=0,$$

which is used for solving the tensile and compression section height, i.e., $h_{1}$ and $h_{2}$.

The bending moment acting on any section is M(x)=M, this will give

(18) $$\int_{0}^{h_{1}}\sigma_{x}^{+}bzdz+\int_{-h_{2}}^{0}\sigma_{x}^{-}bzdz=M.$$

We have, after substituting Equations (12) and (13) into Equation (18)

(19) $$\int_{0}^{h_{1}}\frac{k_{0}b}{\rho }e^{-\alpha_{1}z/h}z^{2}dz+\int_{-h_{2}}^{0}\frac{k_{0}b}{\rho }e^{-\alpha_{2}z/h}z^{2}dz=M.$$

If we let

(20) $$\left\{ \begin{array}{l} \int_{0}^{h_{1}}e^{-\alpha_{1}z/h}z^{2}dz=e^{-\alpha_{1}h_{1}/h}\left(-\frac{hh_{1}^{2}}{\alpha_{1}}-\frac{2h^{2}h_{1}}{\alpha_{1}^{2}}-\frac{2h^{3}}{\alpha_{1}^{3}}\right)+\frac{2h^{3}}{\alpha_{1}^{3}}=A_{2}^{+}\\ \int_{-h_{2}}^{0}e^{-\alpha_{2}z/h}z^{2}dz=e^{\alpha_{2}h_{2}/h}\left(\frac{hh_{2}^{2}}{\alpha_{2}}-\frac{2h^{2}h_{2}}{\alpha_{2}^{2}}+\frac{2h^{3}}{\alpha_{2}^{3}}\right)-\frac{2h^{3}}{\alpha_{2}^{3}}=A_{2}^{-} \end{array}\right.,$$

thus from Equation (19), we have a familiar form concerning deformation as follows

(21) $$\frac{1}{\rho }=\frac{M}{k_{0}b(A_{2}^{+}+A_{2}^{-})}=\frac{M}{D^{*}},$$

where $D^{*}$ is defined as the bending stiffness of a bimodular FGPM beam, $D^{*}=k_{0}b(A_{2}^{+}+A_{2}^{-})$. Substituting Equation (21) into Equations (12) and (13), we have, for $0\leq z\leq h_{1}$,

(22) $$\sigma_{x}^{+}=\frac{M}{b(A_{2}^{+}+A_{2}^{-})}ze^{-\alpha_{1}z/h},$$

and for $-h_{2}\leq z\leq 0$,

(23) $$\sigma_{x}^{-}=\frac{M}{b(A_{2}^{+}+A_{2}^{-})}ze^{-\alpha_{2}z/h}.$$

If we let the deflection be *w*, Euler-Bernoulli equation in small-deflection case will give

(24) $$\frac{1}{\rho }=-\frac{d^{2}w}{dx^{2}}=\frac{M}{k_{0}b(A_{2}^{+}+A_{2}^{-})}.$$

Integrating the above equation with respect to *x* will yield (*M* is a constant)

(25) $$w(x)=-\frac{M}{2k_{0}b(A_{2}^{+}+A_{2}^{-})}x^{2}+cx+d,$$

where *c* and *d* are two integrating constants and may be determined by the following boundary conditions:

(26) $$w=\frac{dw}{dx}=0,\;\mathrm{at}\;x=l.$$

Thus,

(27) $$c=\frac{Ml}{k_{0}b(A_{2}^{+}+A_{2}^{-})},d=\frac{-Ml^{2}}{2k_{0}b(A_{2}^{+}+A_{2}^{-})}.$$

Substituting it into Equation (25), we finally obtain

(28) $$w(x)=-\frac{M}{2k_{0}b(A_{2}^{+}+A_{2}^{-})}{\left(x-l\right)}^{2}.$$

For the convenience of the next comparison, when the cantilever beam is subjected a uniformly-distributed load on its upper surface, $M(x)=qx^{2}/2$, the two integrating constants may be again determined as

(29) $$c=\frac{ql^{3}}{6k_{0}b(A_{2}^{+}+A_{2}^{-})},d=-\frac{ql^{4}}{8k_{0}b(A_{2}^{+}+A_{2}^{-})}.$$

Thus, we have

(30) $$w(x)=\frac{q}{24k_{0}b(A_{2}^{+}+A_{2}^{-})}(-x^{4}+4l^{3}x-3l^{4}).$$

### 3.2. Electrical Displacement

Next, we will derive the electrical displacement components $D_{x}^{+/-}$ in one-dimensional case. Note that the electrical displacement is generated not only from the electrical voltage application in piezoelectric elements, as indicated in actuator model, but also from the mechanical load, as indicated in sensor model which agrees with our study model, according to Figure 2 (under the action of bending moment).

From Equations (1), (6), (22) and (23), we have

(31) $$E_{z}^{+/-}=-\frac{d_{31}^{0}e^{\alpha_{i}z/h}}{\lambda_{33}^{0}e^{\alpha_{i}z/h}}\frac{M}{b(A_{2}^{+}+A_{2}^{-})}ze^{-\alpha_{i}z/h}=-\frac{d_{31}^{0}}{\lambda_{33}^{0}}\frac{M}{b(A_{2}^{+}+A_{2}^{-})}ze^{-\alpha_{i}z/h}=-\frac{l_{0}M}{b(A_{2}^{+}+A_{2}^{-})}ze^{-\alpha_{i}z/h},$$

where $l_{0}=d_{31}^{0}/\lambda_{33}^{0}$. Let the potential function be $\Phi^{+/-}$, the relation of electrical field and potential in two-dimensional problem will give

(32) $$E_{x}^{+/-}=-\frac{\partial \Phi^{+/-}}{\partial x},E_{z}^{+/-}=-\frac{\partial \Phi^{+/-}}{\partial z}.$$

Obviously, if $E_{z}^{+/-}$ is known, $\Phi^{+/-}=-\int E_{z}^{+/-}dz+f(x)$ where f(x) is an unknown function concerning only *x*. However, in the one-dimensional problem, we think the variation of $\Phi^{+/-}$ with *x* is only embodied in the bending moment which has been included in $E_{z}^{+/-}$ (see Equation (31)). This fact may be further demonstrated, from the side, based on the previous work [31] concerning purely piezoelectric materials without bimodular functionally graded properties, in which the potential function was determined as, in a two-dimensional case [31],

(33) $$\Phi (x,z)=\left(\frac{h^{2}}{8}-\frac{z^{2}}{2}\right)\frac{d_{31}}{\lambda_{33}}\frac{P}{I}x,$$

where *I* is the moment of inertia of cross section, *P* is the concentrated force acting on the left end of the beam thus Px stands for the bending moment. Obviously, f(x) in the integration $\Phi^{+/-}=-\int E_{z}^{+/-}dz+f(x)$ may be neglected, thus $\Phi^{+/-}=-\int E_{z}^{+/-}dz$ holds. Accordingly, we have

(34) $$\Phi^{+/-}=-\int_{0}^{z}E_{z}^{+/-}dz=\frac{l_{0}M}{b(A_{2}^{+}+A_{2}^{-})}\int_{0}^{z}ze^{-\alpha_{i}z/h}dz=\frac{l_{0}M}{b(A_{2}^{+}+A_{2}^{-})}\left[e^{-\alpha_{i}z/h}\left(-\frac{hz}{\alpha_{i}}-\frac{h^{2}}{\alpha_{i}^{2}}\right)+\frac{h^{2}}{\alpha_{i}^{2}}\right].$$

Substituting Equation (34) into the first expression of Equation (32) and also noting $M(x)=qx^{2}/2$ if uniformly-distributed load is still considered here, $E_{x}^{+/-}$ may be determined as, for $0\leq z\leq h_{1}$,

(35) $$E_{x}^{+}=-\frac{\partial \Phi^{+}}{\partial x}=\frac{l_{0}qx}{b(A_{2}^{+}+A_{2}^{-})}\left[e^{-\alpha_{1}z/h}\left(\frac{hz}{\alpha_{1}}+\frac{h^{2}}{\alpha_{1}^{2}}\right)-\frac{h^{2}}{\alpha_{1}^{2}}\right],$$

and for $-h_{2}\leq z\leq 0$,

(36) $$E_{x}^{-}=-\frac{\partial \Phi^{-}}{\partial x}=\frac{l_{0}qx}{b(A_{2}^{+}+A_{2}^{-})}\left[e^{-\alpha_{2}z/h}\left(+\frac{hz}{\alpha_{2}}+\frac{h^{2}}{\alpha_{2}^{2}}\right)-\frac{h^{2}}{\alpha_{2}^{2}}\right].$$

From the first expression of Equation (5), $D_{x}^{+/-}$ is, for $0\leq z\leq h_{1}$,

(37) $$D_{x}^{+}=\lambda_{11}^{+}E_{x}^{+}=\frac{\lambda_{11}^{0}l_{0}qx}{b(A_{2}^{+}+A_{2}^{-})}\left(\frac{hz}{\alpha_{1}}+\frac{h^{2}}{\alpha_{1}^{2}}-e^{\alpha_{1}z/h}\frac{h^{2}}{\alpha_{1}^{2}}\right),$$

and for $-h_{2}\leq z\leq 0$

(38) $$D_{x}^{-}=\lambda_{11}^{-}E_{x}^{-}=\frac{\lambda_{11}^{0}l_{0}qx}{b(A_{2}^{+}+A_{2}^{-})}\left(\frac{hz}{\alpha_{2}}+\frac{h^{2}}{\alpha_{2}^{2}}-e^{\alpha_{2}z/h}\frac{h^{2}}{\alpha_{2}^{2}}\right).$$

Finally, we obtain the bending stress $\sigma_{x}^{+/-}$, the vertical deflection *w* and electrical displacement $D_{x}^{+/-}$ in the one-dimensional problem.

## 4. Two-Dimensional Numerical Simulation

**Figure 3:**
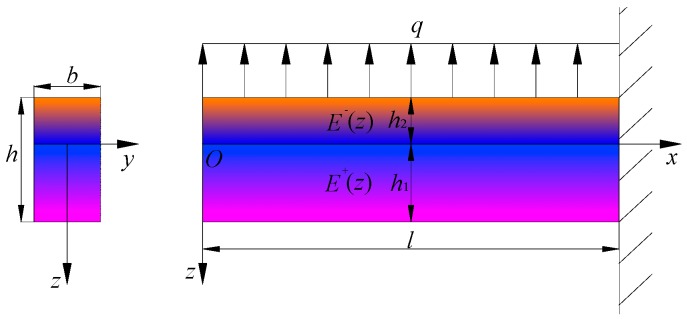
Scheme of a bimodular FGPM cantilever beam under uniformly-distributed load.

In this section we will use the software ABAQUS to simulate a bimodular FGPM cantilever beam subjected to a uniformly-distributed load *q* on its upper surface, as shown in Figure 3, in which other physical quantities and the establishment of coordinate system are the same as those in Section 2 and Section 3. At the same time, it is assumed that 1 corresponds to the *x* direction, 2 to the *y* direction and 3 to the *z* direction.

### 4.1. Constitutive Equation of Piezoelectrical Materials

In ABAQUS, e-form constitutive equation of piezoelectrical materials is adopted, such that

(39) $$\left\{ \begin{array}{l} \sigma_{ij}=D_{ijkl}^{E}\varepsilon_{kl}-e_{mij}^{\varphi }E_{m}\\ q_{ij}=e_{mij}^{\varphi }\varepsilon_{jk}+D_{ij}^{\varphi \left(\varepsilon \right)}E_{j} \end{array}\right.\left(i,j,k,l,m=1,\;2,\;3\right),$$

where $\sigma_{ij}$ is the stress component; $\varepsilon_{ij}$ is the strain component; $q_{ij}$ is the electrical displacement component; $D_{ijkl}^{E}$ is the stiffness coefficient matrix; $D_{ij}^{\varphi (\varepsilon )}$ is the piezoelectrical stress constants matrix; $e_{mij}^{\varphi }$ is the dielectric constant matrix; $E_{m}$ and $E_{j}$ are electrical field strength.

The *z* axis is set to be the polarization direction, corresponding to the 3-direction of ABAQUS. Since the elastic properties of ABAQUS are represented by the stiffness coefficient matrix $D_{ijkl}$ of corresponding material, which is the inverse matrix of the flexibility matrix, it is necessary to transform the flexibility coefficient matrix of the above material into the stiffness coefficient matrix, i.e.,

(40) $$\left[s_{ij}\right]=\left[\begin{array}{cccccc} s_{11} & s_{12} & s_{13} & 0 & 0 & 0\\ s_{21} & s_{22} & s_{23} & 0 & 0 & 0\\ s_{31} & s_{32} & s_{33} & 0 & 0 & 0\\ 0 & 0 & 0 & s_{66} & 0 & 0\\ 0 & 0 & 0 & 0 & s_{44} & 0\\ 0 & 0 & 0 & 0 & 0 & s_{44} \end{array}\right]\Rightarrow \left[c_{ij}\right]=\left[\begin{array}{cccccc} c_{11} & c_{12} & c_{13} & 0 & 0 & 0\\ c_{21} & c_{22} & c_{23} & 0 & 0 & 0\\ c_{31} & c_{32} & c_{33} & 0 & 0 & 0\\ 0 & 0 & 0 & c_{66} & 0 & 0\\ 0 & 0 & 0 & 0 & c_{44} & 0\\ 0 & 0 & 0 & 0 & 0 & c_{44} \end{array}\right].$$

Note that since we define the variation form of functionally graded materials in advance, the flexibility coefficient $s_{ij}=s_{ij}^{0}e^{\alpha_{i}z/h}$, where i=1,2 due to the bimodular effect, thus, the stiffness coefficient will change as $c_{ij}=c_{ij}^{0}e^{-\alpha_{i}z/h}$, otherwise $s_{ij}$ and $c_{ij}$ cannot satisfy $\left[s_{ij}][c_{ij}]=[s_{ij}^{0}]e^{\alpha_{i}z/h}[c_{ij}^{0}]e^{-\alpha_{i}z/h}=[E\right]$, where [E] is an unit matrix. At the same time, piezoelectrical strain constants $e_{ij}=d_{ij}c_{ij}^{E}=d_{ij}^{0}e^{\alpha_{i}z/h}c_{ij}^{0}e^{-\alpha_{i}z/h}=e_{ij}^{0}$, where $c^{E}$ is short circuit elastic stiffness constant matrix, thus piezoelectrical stress constants matrix is

(41) $$\left[e_{ij}\right]=\left[e_{ij}^{0}\right]=\left[\begin{array}{cccccc} 0 & 0 & 0 & 0 & e_{15} & 0\\ 0 & 0 & 0 & 0 & 0 & e_{15}\\ e_{31} & e_{31} & e_{33} & 0 & 0 & 0 \end{array}\right].$$

The constitutive equation of piezoelectric materials is expressed as follows, in the form of matrix,

(42) $$\left[\begin{array}{c} \sigma_{11}\\ \sigma_{22}\\ \sigma_{33}\\ \sigma_{12}\\ \sigma_{13}\\ \sigma_{23} \end{array}\right]=\left[\begin{array}{cccccc} c_{11} & c_{12} & c_{13} & 0 & 0 & 0\\ c_{12} & c_{11} & c_{13} & 0 & 0 & 0\\ c_{13} & c_{13} & c_{33} & 0 & 0 & 0\\ 0 & 0 & 0 & c_{66} & 0 & 0\\ 0 & 0 & 0 & 0 & c_{44} & 0\\ 0 & 0 & 0 & 0 & 0 & c_{44} \end{array}\right]\left[\begin{array}{c} \varepsilon_{11}\\ \varepsilon_{22}\\ \varepsilon_{33}\\ \gamma_{12}\\ \gamma_{13}\\ \gamma_{23} \end{array}\right]-\left[\begin{array}{ccc} 0 & 0 & e_{31}\\ 0 & 0 & e_{31}\\ 0 & 0 & e_{33}\\ 0 & 0 & 0\\ e_{15} & 0 & 0\\ 0 & e_{15} & 0 \end{array}\right]\left[\begin{array}{c} E_{1}\\ E_{2}\\ E_{3} \end{array}\right]$$

and

(43) $$\left[\begin{array}{c} D_{1}\\ D_{2}\\ D_{3} \end{array}\right]=\left[\begin{array}{cccccc} 0 & 0 & 0 & 0 & e_{15} & 0\\ 0 & 0 & 0 & 0 & 0 & e_{15}\\ e_{31} & e_{31} & e_{33} & 0 & 0 & 0 \end{array}\right]\left[\begin{array}{c} \varepsilon_{11}\\ \varepsilon_{22}\\ \varepsilon_{33}\\ \gamma_{12}\\ \gamma_{13}\\ \gamma_{23} \end{array}\right]+\left[\begin{array}{ccc} \lambda_{11} & 0 & 0\\ 0 & \lambda_{11} & 0\\ 0 & 0 & \lambda_{33} \end{array}\right]\left[\begin{array}{c} E_{1}\\ E_{2}\\ E_{3} \end{array}\right].$$

In ABAQUS, the double-subscript second-order tensor mark, 11, 22, 33, 13, 23 and 12, correspond to the vector components, 1, 2, 3, 4, 5 and 6, respectively. Thus, the above two equations are expressed as, in ABAQUS

(44) $$\left[\begin{array}{c} \sigma_{11}\\ \sigma_{22}\\ \sigma_{33}\\ \sigma_{12}\\ \sigma_{13}\\ \sigma_{23} \end{array}\right]=\left[\begin{array}{cccccc} D_{1111} & D_{1122} & D_{1133} & 0 & 0 & 0\\ D_{2211} & D_{2222} & D_{2233} & 0 & 0 & 0\\ D_{3311} & D_{3322} & D_{3333} & 0 & 0 & 0\\ 0 & 0 & 0 & D_{1212} & 0 & 0\\ 0 & 0 & 0 & 0 & D_{1313} & 0\\ 0 & 0 & 0 & 0 & 0 & D_{1313} \end{array}\right]\left[\begin{array}{c} \varepsilon_{11}\\ \varepsilon_{22}\\ \varepsilon_{33}\\ \gamma_{12}\\ \gamma_{13}\\ \gamma_{23} \end{array}\right]-\left[\begin{array}{ccc} 0 & 0 & e_{311}\\ 0 & 0 & e_{322}\\ 0 & 0 & e_{333}\\ 0 & 0 & 0\\ e_{113} & 0 & 0\\ 0 & e_{223} & 0 \end{array}\right]\left[\begin{array}{c} E_{1}\\ E_{2}\\ E_{3} \end{array}\right]$$

and

(45) $$\left[\begin{array}{c} q_{1}\\ q_{2}\\ q_{3} \end{array}\right]=\left[\begin{array}{cccccc} 0 & 0 & 0 & 0 & e_{113} & 0\\ 0 & 0 & 0 & 0 & 0 & e_{113}\\ e_{311} & e_{322} & e_{333} & 0 & 0 & 0 \end{array}\right]\left[\begin{array}{c} \varepsilon_{11}\\ \varepsilon_{22}\\ \varepsilon_{33}\\ \gamma_{12}\\ \gamma_{13}\\ \gamma_{23} \end{array}\right]+\left[\begin{array}{ccc} D_{11} & 0 & 0\\ 0 & D_{11} & 0\\ 0 & 0 & D_{33} \end{array}\right]\left[\begin{array}{c} E_{1}\\ E_{2}\\ E_{3} \end{array}\right],$$

where, $D_{ijkl}$ is the modulus of elasticity, $D_{ij}$ is the dielectric coefficient and $q_{i}$ is the electrical displacement component. Through the comparison of the above two sets of equations, it is easy to see the corresponding relationship of constants, which can be used to input values of the constants. For example, we should input $c_{11}$ at the location of $D_{1111}$ in ABAQUS; input $e_{31}$ at the location of $e_{311}$ and input $\lambda_{11}$ at the location of $D_{11}$.

### 4.2. Modeling and Simulation

ABAQUS software is one of the large-scale finite element software at present which can analyze complex engineering mechanics problems including the problem of piezoelectric materials. However, the software itself does not involve different properties in tension and compression and functionally graded properties of materials varying in a form of continuous function. For this purpose, we should resort to subareas model in tension-compression and layer-wise theory to simulate the problem studied, which inevitably complicates the analysis process further. The detailed steps for modeling and simulation are as follows.

(i) Establishment of entity structure

The solid model of a FGPM cantilever beam is established, in which the length of the beam *l* is set to be 1 m, the section height *h* to be 0.2 m and the section width *b* to be 0.08 m.

(ii) Determination of neutral layer and tension-compression subarea

**Figure 4:**
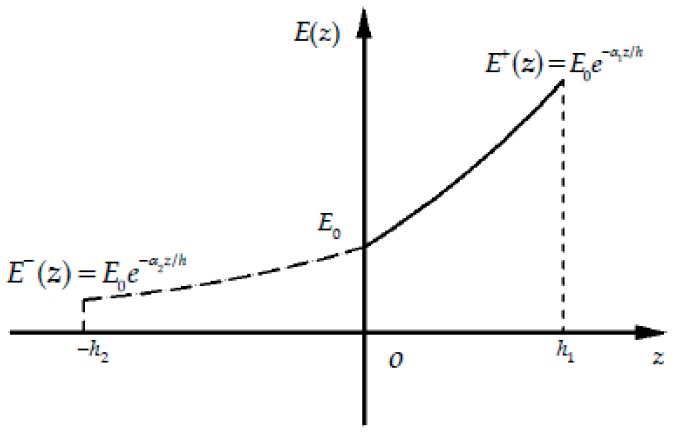
$E^{+}(z)$ and $E^{-}(z)$ when $\alpha_{1}=-2$ and $\alpha_{2}=-3$.

Note that comparing to common piezoelectric cantilever beam, the subarea of tension and compression of beam under external loads is a newly introduced feature, we must determine the unknown neutral layer first, thus realizing the so-called tension-compression subarea. For this purpose, a set of functionally graded indexes should be chosen to determine the tensile height and the compressive one of the beams, according to the one-dimensional theoretical solution. For example, consider the case $\alpha_{1}=-2$ and $\alpha_{2}=-3$. Substituting the given values into Equations (16) and (17) and also noting the section total height *h* = 0.2 m, we may have *h*_1_ = 0.06 m and *h*_2_ = 0.14 m. It is easy to see that due to the tensile modulus $E^{+}(z)=E_{0}e^{-\alpha_{1}z/h}$ is totally greater than the compressive modulus $E^{-}(z)=E_{0}e^{-\alpha_{2}z/h}$, the neutral layer will locate below the geometrical middle surface, i.e., $h_{1}<h_{2}$, see Figure 4, in which $E_{0}$ is the modulus of the neutral layer.

**Figure 5:**
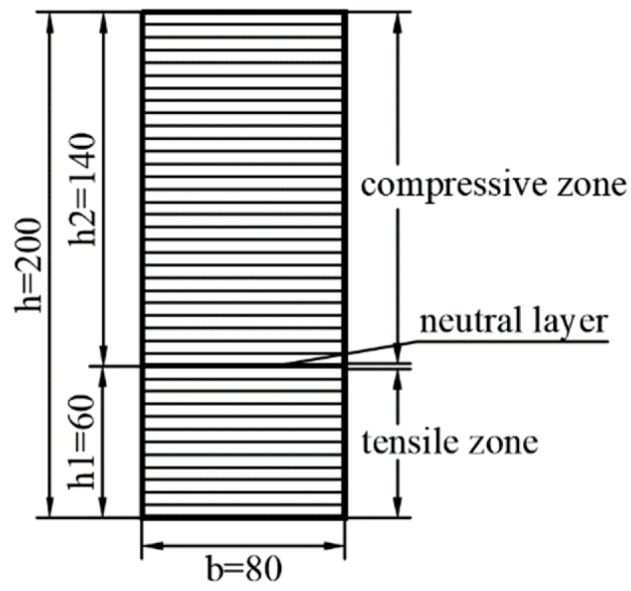
Sketch of layering on cross section of a bimodular FGPM cantilever beam (unit: mm).

As indicated above, the change of material properties as certain direction in ABAQUS cannot be defined as a continuous function, therefore according to conventional practice, we adopt layer-wise model to simulate the functionally graded properties of the materials. Without losing the computational accuracy, the beam is divided into a moderate number of layers; the physical parameters of the material in each layer are regarded as the same, thus indirectly realizing the continuous change of materials properties as the thickness direction if the layer numbers are enough. For this purpose, bounded by the neutral layer, the upper part and the lower part of the beam are divided equally, thus the beam is divided into 40 layers along the thickness direction, with each layer 5 mm thick, as shown in Figure 5. It is easy to see that there are 12 layers in the tensile zone and 28 layers in the compressive zone. Note that the coordinate origin now locates at the neutral layer.

(iii) Determination of properties of materials

The material constant at the neutral layer z=0 is shown in Table 1, which may be input into ABAQUS directly. Material constants on other layers which do not locate at the neutral layer may be computed and input into the program, via the layering model of tension-compression subarea established in Step (ii). 

(iv) Establishment of boundary conditions

The left end of the beam is free and the right end is fully fixed, which agrees with the mechanical model shown in Figure 3. For this purpose, we need to define the fixed constrain on the right end of the cantilever beam in ABAQUS, including displacement and rotation.

(v) Mesh division

In this simulation, an 8-node linear piezoelectric brick C3D8E is adopted, in which the size ratio is set to be 0.01 and the mesh size is 5 mm × 10 mm. C3D8E may well realize the simulation of a cantilever beam under the mechanical load and electrical load.

(vi) Step module and adding loads

An analysis step named Static General is established to apply the load. In this simulation, uniformly-distributed load form is considered only. For this purpose, a uniformly-distributed load (*q* = 1 N/m^2^), along the negative direction of *z* axis, is applied on the upper surface of the beam.

Up to now, the modeling job has been finished. We note that in the above steps, the difference introduced by bimodular functionally graded properties embodies mainly in Steps (ii) and (iii), which makes the analysis more complicated. 

(vii) Operation and results output

**Figure 6:**
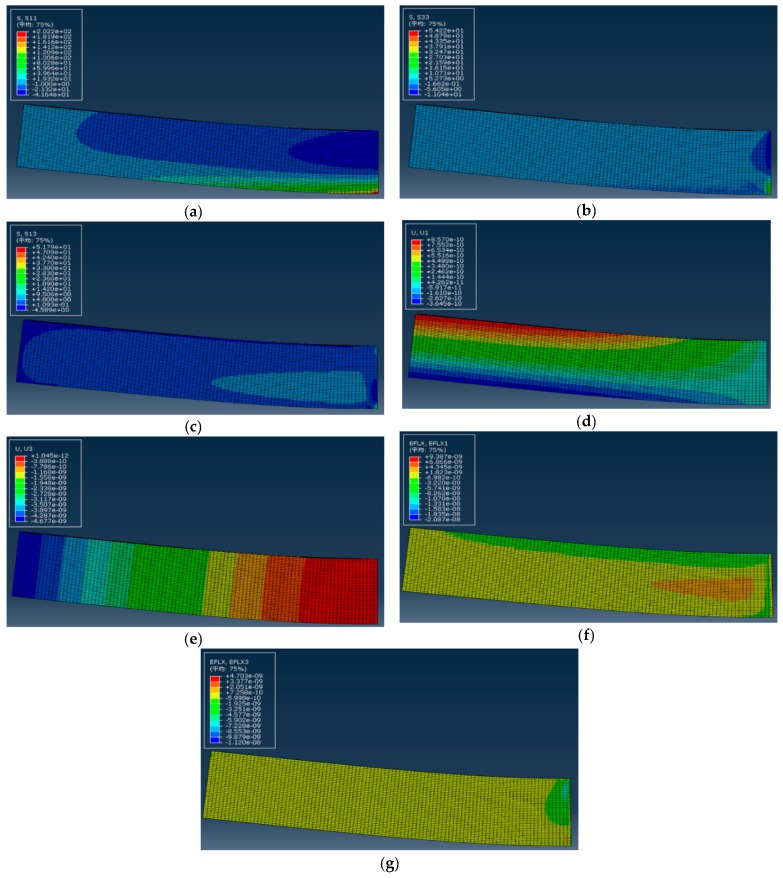
Cloud diagrams of mechanical stresses and displacements, and electrical displacements: (**a**) The cloud diagram of $\sigma_{x}$; (**b**) The cloud diagram of $\sigma_{z}$; (**c**) The cloud diagram of $\tau_{xz}$; (**d**) The cloud diagram of u; (**e**) The cloud diagram of w; (**f**) The cloud diagram of $D_{x}$; (**g**) The cloud diagram of $D_{z}$.

After a job is established, submit the job and calculate and output the results. It should be noted here that although the numerical simulation is based on a three-dimensional case, the problem we study still attributes to two-dimensional plane problem. Therefore, we only output the results concerning plane problem, and meanwhile, it is also convenient to compare these outputs with our previous study [27] which is exactly the case of two-dimensional problem. Figure 6 shows the cloud diagram of the mechanical stresses, $\sigma_{x}$, $\sigma_{z}$ and $\tau_{xz}$, the mechanical displacement, u and w, as well as the electrical displacement, $D_{x}$ and $D_{z}$ in a two-dimensional problem.

## 5. Comparisons and Discussions

### 5.1. Comparison of One-Dimensional Solution and Two-Dimensional Simulation

Due to the fact that in the derivation of one-dimensional theoretical solution, some assumptions have to be introduced to obtain a simple but clear expression, the validity of one-dimensional solution should be further verified. For this purpose, we use the results from the two-dimensional numerical simulation to validate the rationality of the one-dimensional solution.

**Figure 7:**
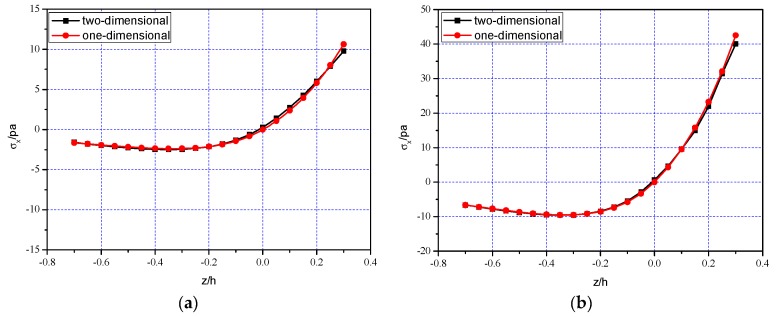

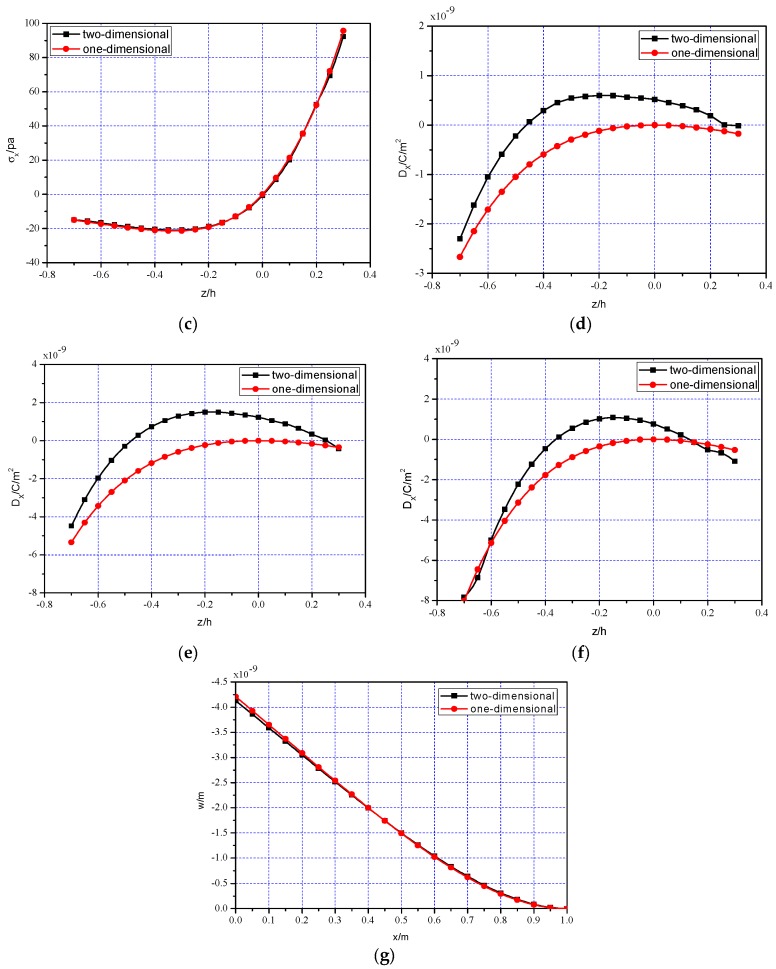
Comparisons of one-dimensional solution and two-dimensional simulation: (a) $\sigma_{x}$ at x=0.25l; (**b**) $\sigma_{x}$ at x=0.5l; (**c**) $\sigma_{x}$ at x=0.75l; (**d**) $D_{x}$ at x=0.25l; (**e**) $D_{x}$ at x=0.5l; (**f**) $D_{x}$ at x=0.75l; (**g**) w(x) at z=0.

Figure 7 shows the comparison results between one-dimensional theoretical solution and two-numerical numerical simulation. Figure 7a–c and Figure 7d–f show that the bending stress $\sigma_{x}$ and the electrical displacement $D_{x}$ at different cross sections x=0.25l, 0.5l, 0.75l vary with the thickness direction, respectively; Figure 7g shows the deflection w(x) at z=0. From Figure 7, it is easy to see that the two solutions curves are roughly close to each other, which indicates the validity of one-dimensional theoretical solution, to some extent. Besides, Table 2 and Table 3 further show the relative errors of the one-dimensional solution and two-dimensional simulation concerning $\sigma_{x}$ at x=0.25l and the vertical deflection w(x), respectively. It is obvious that the relative errors are within acceptable limits. Note that in Table 2, the value of ABAQUS simulation at the neutral layer z/h=0 gives 0.2813 but zero (while the theoretical solution gives zero, according to Equation (11)). This error may be caused by the large discreteness of the finite element calculation itself, especially near the neutral layer. The neutral layer may be regarded as a computing-sensitive zone in which the tensile and compressive stresses change their positive or negative sign at this layer and for bimodular functional graded materials, coefficient of materials is continuous at this layer but their first-order derivative to *z*-direction is not continuous, see our previous study [29]. To obtain more accurate results, there is a need to increase the number of mesh division, especially at the neutral layer, thus enlarging the amount of computation. 

It should be noted here, that since the one-dimensional theoretical solution is derived on a relatively simple case, the number of physical quantities obtained is relatively limited. Due to pure bending, for example, only $\sigma_{x}$ is derived while $\tau_{xz}$ and $\sigma_{z}$ cannot be obtained in this way; similarly, only the deflection w(x) may be obtained in the one-dimensional solution while the so-called horizontal displacement *u* cannot be considered.

### 5.2. Comparison of Two-Dimensional Numerical Simulation and Existing Solution

He et al. [27] derived a two-dimensional theoretical solution for FGPM cantilever beam with bimodular effect under combined loads. Therefore, it is interesting to compare the two-dimensional numerical results presented in this study with the existing solution from He et al. [27].

**Figure 8:**
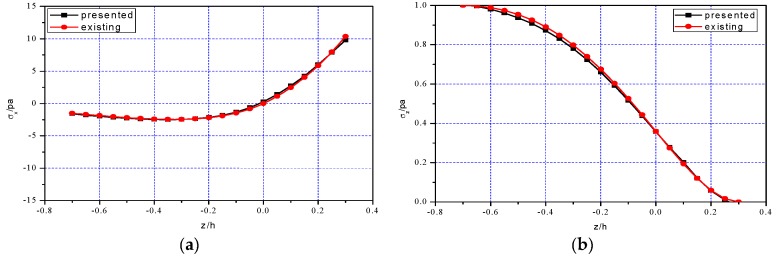

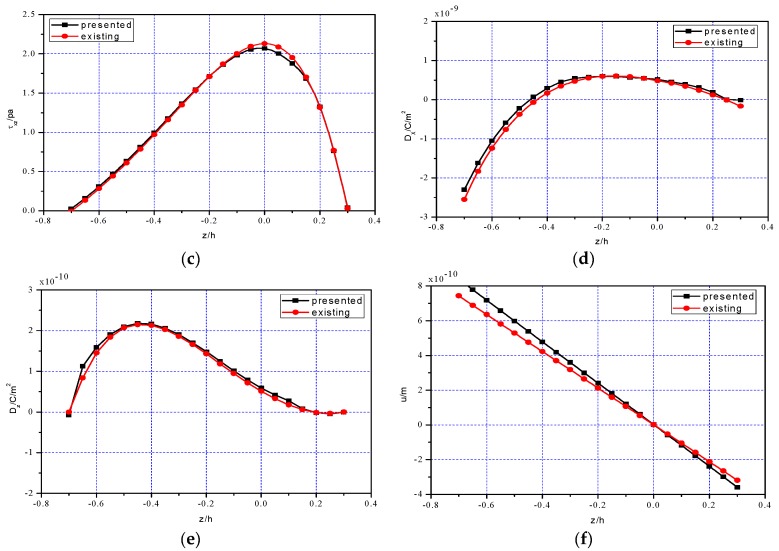
Comparisons of two-dimensional simulation presented in this study and existing solution from He et al. [27] at x=0.25l: (**a**) Stress $\sigma_{x}$; (**b**) Stress $\sigma_{z}$; (**c**) Stress $\tau_{xz}$; (**d**) Electrical displacement $D_{x}$; (**e**) Electric displacement $D_{z}$; (**f**) Mechanical displacement u.

**Figure 9:**
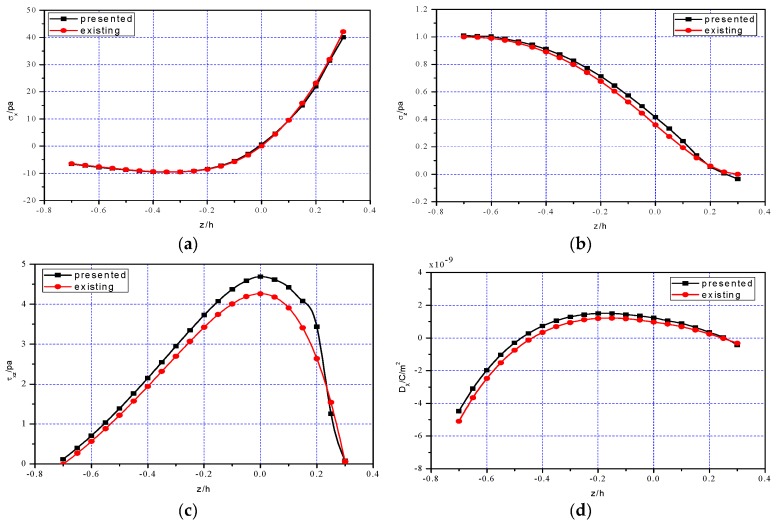

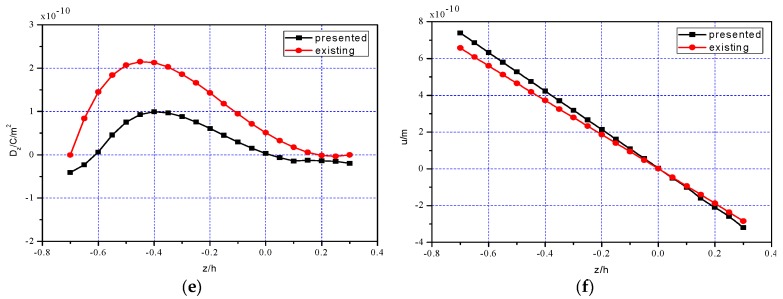
Comparisons of two-dimensional simulation presented in this study and existing solution from He et al. [27] at x=0.5l: (**a**) Stress $\sigma_{x}$; (**b**) Stress $\sigma_{z}$; (**c**) Stress $\tau_{xz}$; (**d**) Electrical displacement $D_{x}$; (**e**) Electric displacement $D_{z}$; (**f**) Mechanical displacement u.

**Figure 10:**
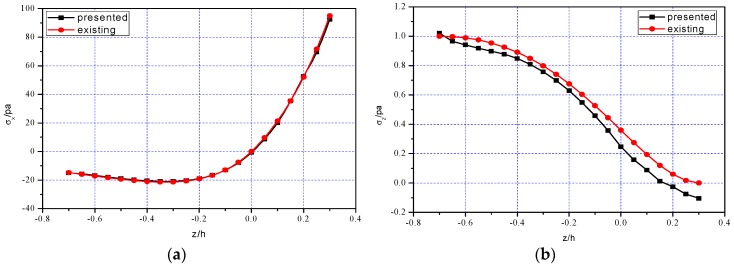

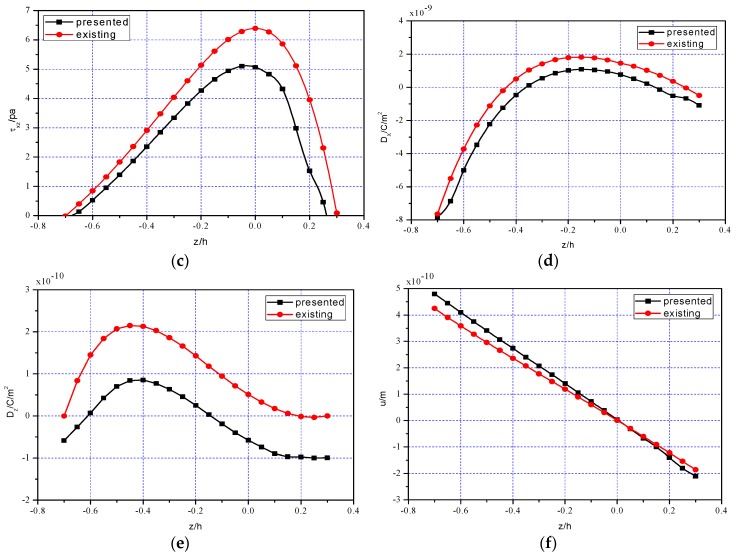
Comparisons of two-dimensional simulation presented in this study and existing solution from He et al. [27] at x=0.75l: (**a**) Stress $\sigma_{x}$; (**b**) Stress $\sigma_{z}$; (**c**) Stress $\tau_{xz}$; (**d**) Electrical displacement $D_{x}$; (**e**) Electric displacement $D_{z}$; (**f**) Mechanical displacement u.

Figure 8, Figure 9 and Figure 10 show the comparison results of the two-dimensional numerical simulation and the existing solution, in which the computational data of the existing solution are from [27]. Table 4, Table 5 and Table 6 further show the relative errors of the two-dimensional simulation presented in this study and existing work [27], in which $\sigma_{x}$, $\tau_{xz}$, u, $D_{x}$ and $D_{z}$ at x=0.25l are considered. The results show that the two solutions are approximately equal, and the relative error is also in the acceptable range, excluding a few points.

From the above results, it is easy to see that among the two-dimensional mechanical physical quantities, stresses $\sigma_{x}$, $\tau_{xz}$ and $\sigma_{z}$ as well as displacements u and w, their importance is different. For stress components, the bending stress $\sigma_{x}$ is still the dominant stress, the shear stress $\tau_{xz}$ is secondary and $\sigma_{z}$ is almost negligible; for displacement components, it is obvious that only w is the interesting variables in our analysis while u is negligible small. This conclusion is consistent with the existing results. Similarly, among the two-dimensional electrical physical quantities, for example, $D_{x}$ and $D_{z}$, the final result indicates $D_{x}>D_{z}$ and in some cases even $D_{x}>>D_{z}$, this fact may well explain the rationality of the assumption $D_{z}\approx 0$ in the derivation of one-dimensional theoretical solution.

In addition, it should be noted here that, from Figure 9e and Figure 10e, we may find that $D_{z}$ obtained by the two solutions have obvious difference, and for some points the maximum relative error is even greater than 50%. However, comparing with $D_{x}$ having the same attribute, $D_{z}$ is an unimportant quantity, as indicated above. Therefore, even if there is slightly big difference in $D_{z}$, its influence on the whole problem is relatively limited, this is the reason why we obtained the one-dimensional theoretical solution by neglecting $D_{z}$. Since the quantity itself can be ignored, the difference of this quantity is even more insignificant.

### 5.3. Comparison of One-Dimensional Theoretical Solution and Existing Solutions

In existing studies, Shi and Chen [17] gave a set of analytical solutions for the problem of a FGPM cantilever beam subjected to different loadings, and Yang and Liu [31] derived the solution for the problem of a piezoelectric cantilever beam under an end load. In this section, we will compare the one-dimensional solution derived in this paper with the two solutions mentioned above.

Before comparison, it is necessary to degrade these solutions into a one-dimensional case since they were obtained based on different conditions. For example, in [17], the effect of body force $F_{z}$ is considered, and only the elastic parameter $S_{33}$ changes along the *z* direction; while in this study, the different properties in tension and compression are taken into account, and all the material parameters change along the *z* direction. For the convenience of comparison, we let the body force $F_{z}$ from [17] be zero, and the elastic parameter $S_{33}$ from [17] be a constant, that is,

(46) $$m_{1}=0,m_{2}=0,m_{3}=0,m_{4}=0,r_{1}=0,r_{2}=0,F=-P.$$

where the meaning of these quantities above can be found in [17]. At the same time, we redefine some quantities from Section 3 as follows

(47) $$\alpha_{1}=\alpha_{2}=0,\;and\;M=-Px.$$

Thus, the problem solved in this study and the problem in [17] are now degraded to the problem of a piezoelectric cantilever beam under an end load, that is, the problem solved in [31]. The detailed degradation is shown as follows.

Firstly, let us degrade the solution obtained in [17]. Substituting Equation (46) into the Equations (14) and (17) in [17], we may obtain

(48) $$\begin{array}{l} d^{*}=0,a_{1}=0,a_{3}=0,a_{4}=0,b_{2}=-\frac{2P}{h^{3}},b_{5}=0,b_{6}=\frac{3P}{2h},d_{1}=0,d_{2}=0,\\ d_{3}=0,d_{4}=0,A_{1}=0,A_{3}=0,A_{4}=0,B_{2}=-\frac{2gP}{h^{3}},B_{5}=0,B_{6}=\frac{gP}{2h} \end{array}$$

From Equation (48) and the Equation (11) in [17], it can be obtained that

(49) $$R_{1}=0,R_{2}=a_{2}S_{33}-A_{2}g_{33},R_{3}=0,R_{4}=0,M_{3}=0,Q_{x3}=0.$$

Thus, from Equations (48) and (49), the Equation (8) in [17] can be written as

(50) $${\sigma^{\prime }}_{x}=-\frac{12P}{h^{3}}xz$$

where “′” represents the corresponding quantity derived from [17]. From Equations (48) and (49) as well as the Equation (13) in [17], we have

(51) $$w^{\prime }(x)=\frac{1}{3}R_{2}z^{3}-\frac{6P}{h^{3}}S_{13}z^{2}x+\frac{2P}{h^{3}}S_{11}(x^{3}-3L^{2}x+2L^{3}).$$

When z=0, the deflection equation at the axis of the piezoelectric cantilever beam can be obtained

(52) $$w^{\prime }(x)=\frac{2P}{h^{3}}S_{11}(x^{3}-3L^{2}x+2L^{3}).$$

Next, let us degrade the one-dimensional solution derived in this study. Substituting Equation (47) into Equation (9), we have

(53) $$\sigma_{x}=\frac{\lambda_{33}}{s_{11}\lambda_{33}-{\left(d_{31}\right)}^{2}}\frac{z}{\rho }.$$

Since $\alpha_{1}=\alpha_{2}=0$, we may obtain

(54) $$h_{1}=h_{2}=h/2.$$

The integrations in Equation (20) may be simplified as

(55) $$\left\{ \begin{array}{l} A_{2}^{+}=\int_{0}^{h/2}z^{2}dz=\frac{h^{3}}{24}\\ A_{2}^{-}=\int_{-h/2}^{0}z^{2}dz=\frac{h^{3}}{24} \end{array}\right..$$

From Equations (55) and (24), we obtain

(56) $$\frac{1}{\rho }=-\frac{d^{2}w}{dx^{2}}=[s_{11}-\frac{{\left(d_{31}\right)}^{2}}{\lambda_{33}}]\frac{12M}{bh^{3}}=-[s_{11}-\frac{{\left(d_{31}\right)}^{2}}{\lambda_{33}}]\frac{12P}{bh^{3}}x.$$

Integrating the above equation with respect to *x* will yield

(57) $$w=[s_{11}-\frac{{\left(d_{31}\right)}^{2}}{\lambda_{33}}]\frac{2Px^{3}}{bh^{3}}+cx+d.$$

From Equation (26), we may obtain

(58) $$c=-[s_{11}-\frac{{\left(d_{31}\right)}^{2}}{\lambda_{33}}]\frac{6Pl^{2}}{bh^{3}},d=[s_{11}-\frac{{\left(d_{31}\right)}^{2}}{\lambda_{33}}]\frac{4Pl^{3}}{bh^{3}}.$$

Thus

(59) $$w=\frac{2P}{bh^{3}}[s_{11}-\frac{{\left(d_{31}\right)}^{2}}{\lambda_{33}}](x^{3}-3l^{2}x+2l^{3}).$$

Substituting Equation (56) into Equation (53), we finally obtain

(60) $$\sigma_{x}=-\frac{12P}{bh^{3}}xz.$$

By comparing the above equation with Equation (50), it is easy to see that they are identical except the beam width *b*, which is considered to be unit 1 in [17]. 

In addition, from Equations (50) and (60), it is easy to find that $\sigma_{x}$ is the same as the corresponding expression, i.e., the Equation (29) in [31]. For the deflection w, Equation (59) in this study and the Equation (38) in [31] are identical, only with a slight difference in Equation (52). We note that $s_{11}\gg {\left(d_{31}\right)}^{2}/\lambda_{33}$ for the piezoelectric materials PZT-4, as indicated in Section 3.1, thus $s_{11}-{\left(d_{31}\right)}^{2}/\lambda_{33}\approx s_{11}$. From Equations (52) and (59), we may find, $w^{\prime }\approx w$. 

### 5.4. Evolution for One-Dimensional Theoretical Solution

For the one-dimensional solution of beams with different properties of materials, it is convenient to compare all kinds of solution due to the consistency in form. Among the solutions, we take two important physical quantities as our comparing objects, one is modulus of elasticity of materials, $E^{*}$, and another is bending stiffness of beams, $D^{*}$, since they are closely associated with final solutions. For example, if bending stiffness $D^{*}$ is known, the deflection is also easily determined by the classical Euler-Bernoulli equation in small-deflection case, $\frac{1}{\rho }=\frac{d^{2}w}{dx^{2}}=\frac{M}{D^{*}}$, where $D^{*}$ is associated with the modulus of elasticity $E^{*}$ of materials. For this purpose, Table 7 gives $E^{*}$ and $D^{*}$, step by step, from classical beams to bimodular FGPM beams, via bimodular beam [33] and bimodular FGM beam [29]. Interestingly, this order may be called an evolution of material properties from classical beams to bimodular FGPM beams and, in turn, may also be called a regression from bimodular FGPM beams to classical beams. 

It should be noted here that there is an important difference in $A_{2}^{+}$ and $A_{2}^{-}$ for bimodular FGM beams and bimodular FGPM beams. It is found that the integral function index has more than one negative sign for bimodular FGPM beams, i.e., $e^{\alpha_{i}z/h}$ in bimodular FGM beams is now changed as $e^{-\alpha_{i}z/h}$ in bimodular FGPM beams, this because, for bimodular FGPM beams, the original definition for material properties is based on flexibility coefficient $s_{11}^{+/-}$ but stiffness coefficient $E^{+/-}$, and they satisfy $\;s_{11}^{+/-}=s_{11}^{0}e^{\alpha_{i}z/h}=1/E^{+/-}(z)$, thus generating the negative sign.

### 5.5. Discussion on Flexible FGPM Cantilever Beam

In many electromechanical devices, ultra large deflections of cantilevers are generally needed for the sake of application requirements. For example, Merupo et al. [34] investigated the flexoelectric response in soft polyurethane films and their use for large curvature sensing. More recently, Seveno and Guiffard [35] presented the realization of a cantilever-based PZT thin film deposited onto an ultra-thin aluminum foil as a substrate and showed that a very flexible actuator with low voltage-induced ultra large deflections can be obtained by this method. In the above studies, the large deformation analyses of piezoelectric structures are required, not only for static or dynamic problems but also for sensor or actuator models. Thus, via the one-dimensional solution obtained in this study, the deflection of flexible piezoelectric cantilever beam is easily obtained.

The bending stiffness of bimodular FGPM beams, $D^{*}$, has been derived in the Section 3.1, which gives

(61) $$D^{*}=\frac{\lambda_{33}^{0}}{s_{11}^{0}\lambda_{33}^{0}-{\left(d_{31}^{0}\right)}^{2}}b(A_{2}^{+}+A_{2}^{-}).$$

Here, if a large deflection bending is to be considered, the classical Euler-Bernoulli equation will be

(62) $$\frac{1}{\rho }=\frac{d^{2}w/dx^{2}}{{\left[1+{\left(dw/dx\right)}^{2}\right]}^{3/2}}=\frac{M(x)}{D^{*}},$$

where ρ is still the curvature radius of the cantilever beam and *w* is the deflection. We note that here the curvature expression is mathematically precise and has not been the small curvature case mentioned above which reads $1/\rho =d^{2}w/dx^{2}$. Via our previous studies concerning flexible cantilever beams made of classical materials [36], it is convenient to derive the deflection only by replacing the bending stiffness term $D^{*}$ in the solution obtained. This conclusion shows, from the side, the advantage of one-dimensional theoretical solution in predicting deformation of flexible piezoelectric cantilever structures.

## 6. Concluding Remarks

In this study, we used analytical and numerical methods to investigate a FGPM cantilever beams with different properties in tension and compression, in which one-dimensional theoretical solution was derived and two-dimensional numerical simulation was also performed. We made extensive comparisons to validate the rationality of the one-dimensional solution and two-dimensional numerical simulation in this study. The following main conclusions can be drawn.

(i) In the one-dimensional theoretical solution obtained, the presence of modulus of elasticity $E^{*}$ and bending stiffness $D^{*}$ for a bimodular FGPM beam may clearly describe the piezoelectrical effect on the classical problem without electromechanical coupling. The influences introduced by three important material coefficients, i.e., elastic coefficient, piezoelectric coefficient, and dielectric coefficient are all included in $E^{*}$ and $D^{*}$.

(ii) In the two-dimensional numerical simulation, the layered model of tension-compression subarea opens the possibilities for the realization of numerical technique to the problem of FGPM cantilever beam with bimodular effect, although the software itself does not involve different properties in tension and compression of materials.

(iii) The respective advantages of the two solutions are obvious. Comparing with the complex two-dimensional solution, the simplicity in form and the convenience in use make the one-dimensional solution more advantageous in engineering application, for example, in the stage of preliminary design of energy harvesting/sensing/actuating devices based on piezoelectric effect. At the same time, the two-dimensional solution may serve as an effective reference for the refined analysis in the stage of final design. 

The work proposed in this study is helpful for predicting the mechanical behavior of some electromechanical devices. Especially, if these electromechanical devices are made of piezoelectric polymers, they will display relatively strong bimodular properties in tension and compression, which should be given more attention in the stage of analysis and design. The relative work is in progress.

## Figures and Tables

**Table 1:** Physical properties of PZT-4 materials [32].

Elastic Constant (10^−12^ m^2^·N^−1^)					Piezoelectric Constant (10^−12^ C·N^−1^)			Dielectric Constant (10^−8^ F·m^−1^)	
$s_{11}^{0}$	$s_{12}^{0}$	$s_{13}^{0}$	$s_{33}^{0}$	$s_{44}^{0}$	$d_{31}^{0}$	$d_{33}^{0}$	$d_{15}^{0}$	$\lambda_{11}^{0}$	$\lambda_{33}^{0}$
12.4	−3.98	−5.52	16.1	39.1	−135	300	525	1.301	1.151

**Table 2:** Relative errors of two solutions of $\sigma_{x}$ (x=0.25l).

z/h	ABAQUS Simulation (Pa)	Theoretical Solution (Pa)	Relative Errors %
−0.7	−1.5745	−1.6675	5.58
−0.6	−1.9871	−1.9294	2.99
−0.5	−2.2859	−2.1703	5.33
−0.4	−2.4750	−2.3437	5.60
−0.3	−2.4741	−2.3727	4.27
−0.2	−2.1492	−2.1352	0.66
−0.1	−1.3278	−1.4411	7.86
0.0	0.2813	0	**-**
0.1	2.7493	2.3760	15.71
0.2	5.9989	5.8042	3.35
0.3	9.7701	10.6338	8.12

**Table 3:** Relative errors of two solutions of w(x).

x/l	ABAQUS Simulation (10^−^^9^ m)	Theoretical Solution (10^−^^9^ m)	Relative Errors %
0.0	−4.1261	−4.2083	1.95
0.1	−3.5882	−3.6474	1.62
0.2	−3.0502	−3.0884	1.24
0.3	−2.5167	−2.5364	0.78
0.4	−1.9961	−1.9998	0.19
0.5	−1.4993	−1.4905	0.59
0.6	−1.0408	−1.0235	1.69
0.7	−0.6385	−0.6174	3.42
0.8	−0.3132	−0.2940	6.54
0.9	−0.0896	−0.0787	13.91
1.0	0.0000	0	**-**

**Table 4:** Relative errors of two solutions of $\sigma_{x}$ and $\tau_{xz}$ (x=0.25l).

z/h	$\sigma_{x}$			$\tau_{xz}$		
	ABAQUS (Pa)	Analytical (Pa)	Errors %	ABAQUS (Pa)	Analytical (Pa)	Errors %
−0.7	−1.5745	−1.5004	4.94	0.0257	0.0000	**-**
−0.6	−1.9871	−1.8570	7.01	0.3090	0.2817	9.69
−0.5	−2.2859	−2.1748	5.11	0.6349	0.6086	4.32
−0.4	−2.4760	−2.3987	3.22	0.9928	0.9693	2.42
−0.3	−2.4741	−2.4451	1.19	1.3638	1.3474	1.22
−0.2	−2.1492	−2.1921	1.96	1.7137	1.7110	0.16
−0.1	−1.3278	−1.4598	9.04	1.9818	2.0034	1.08
0.0	0.2813	0	**-**	2.0697	2.1306	2.86
0.1	2.7493	2.4601	11.76	1.8770	1.9534	3.91
0.2	5.9989	5.8575	2.41	1.3238	1.3181	0.43
0.3	9.7701	10.3926	5.99	0.0377	0.0306	23.20

**Table 5:** Relative errors of two solutions of u (x=0.25l).

z/h	ABAQUS (10^−^^10^ m)	Analytical (10^−^^10^ m)	Errors %
−0.7	8.3955	7.4356	12.91
−0.6	7.1834	6.3564	13.01
−0.5	5.9828	5.2900	13.10
−0.4	4.7887	4.2308	13.19
−0.3	3.5975	3.1756	13.29
−0.2	2.4065	2.1214	13.44
−0.1	1.2138	1.0660	13.86
0.0	0.0178	0.0088	**-**
0.1	−1.1818	−1.0522	12.32
0.2	−2.3855	−2.1174	12.66
0.3	−3.5941	−3.1873	12.76

**Table 6:** Relative errors of two solutions of $D_{x}$ and $D_{z}$ (x=0.25l).

z/h	$D_{x}$			$D_{z}$		
	ABAQUS (10^−^^9^ C/m^2^)	Analytical (10^−^^9^ C/m^2^)	Errors %	ABAQUS (10^−^^9^ C/m^2^)	Analytical (10^−^^9^ C/m^2^)	Errors %
−0.7	−2.3014	−2.5480	9.68	−0.0769	0.0000	**-**
−0.6	−1.0508	−1.2423	15.42	1.5915	1.4459	10.07
−0.5	−0.2199	−0.3746	41.30	2.1005	2.0677	1.59
−0.4	0.2929	0.1685	73.83	2.1626	2.1316	1.45
−0.3	0.5455	0.4712	15.77	1.9038	1.8621	2.24
−0.2	0.5982	0.5957	0.42	1.4800	1.4252	3.85
−0.1	0.5651	0.5883	3.94	1.0100	0.9440	6.99
0.0	0.5199	0.4832	7.60	0.5887	0.5098	15.48
0.1	0.3928	0.3416	14.99	0.2730	0.1741	56.81
0.2	0.1905	0.1211	57.31	−0.0166	−0.0157	5.73
0.3	−0.0088	−0.1640	94.63	−0.0074	0.0000	**-**

**Table 7:** Evolution for one-dimensional solution of beams (rectangular section).

Material Types of Beams	Modulus of Elasticity	Bending Stiffness
Classical beams	E=const.	$\frac{1}{12}bh^{3}E$
Bimodular beams [33]	$E^{+},E^{-}=const.$, where $E^{+}\neq E^{-}$	$\frac{b}{3}(E^{+}h_{1}^{3}+E^{-}h_{2}^{3})$
Bimodular FGM beams [29]	$\left\{ \begin{array}{l} E^{+}(z)=E_{0}e^{\alpha_{1}z/h}\\ E^{-}(z)=E_{0}e^{\alpha_{2}z/h} \end{array}\right.$, where $\alpha_{1}\neq \alpha_{2}$ and $E_{0}$ is neutral layer modulus	$E_{0}b(A_{2}^{+}+A_{2}^{-})$, where $\left\{ \begin{array}{l} A_{2}^{+}=\int_{0}^{h_{1}}e^{\alpha_{1}z/h}z^{2}dz\\ A_{2}^{-}=\int_{-h_{2}}^{0}e^{\alpha_{2}z/h}z^{2}dz \end{array}\right.$
Bimodular FGPM beams (this study)	$\begin{array}{ll} E^{*} & =\frac{\lambda_{33}^{+/-}}{s_{11}^{+/-}\lambda_{33}^{+/-}-{\left(d_{31}^{+/-}\right)}^{2}}\\ & =\frac{\lambda_{33}^{0}}{s_{11}^{0}\lambda_{33}^{0}-{\left(d_{31}^{0}\right)}^{2}}e^{-\alpha_{i}z/h} \end{array}$, where $\left\{ \begin{array}{l} s_{11}^{+/-}=s_{11}^{0}e^{\alpha_{i}z/h}=1/E^{+/-}(z)\\ d_{31}^{+/-}=d_{31}^{0}e^{\alpha_{i}z/h}\\ \lambda_{33}^{+/-}=\lambda_{33}^{0}e^{\alpha_{i}z/h} \end{array}\right.(i=1,2\;)$ and $s_{11}^{0},d_{31}^{0},\lambda_{33}^{0}$ are neutral layer modulus	$D^{*}=k_{0}b(A_{2}^{+}+A_{2}^{-})$, where $k_{0}=\frac{\lambda_{33}^{0}}{s_{11}^{0}\lambda_{33}^{0}-{\left(d_{31}^{0}\right)}^{2}}$ and $\left\{ \begin{array}{l} A_{2}^{+}=\int_{0}^{h_{1}}e^{-\alpha_{1}z/h}z^{2}dz\\ A_{2}^{-}=\int_{-h_{2}}^{0}e^{-\alpha_{2}z/h}z^{2}dz \end{array}\right.$
